# Chimeric antigen receptor T cells: a novel therapy for solid tumors

**DOI:** 10.1186/s13045-017-0444-9

**Published:** 2017-03-29

**Authors:** Shengnan Yu, Anping Li, Qian Liu, Tengfei Li, Xun Yuan, Xinwei Han, Kongming Wu

**Affiliations:** 1 Department of Oncology, Tongji Hospital of Tongji Medical College, Huazhong University of Science and Technology, Wuhan, 430030 China; 2 Department of Interventional Radiology, The First Affiliated Hospital of Zhengzhou University, Zhengzhou, 450052 China

**Keywords:** CAR-T cell, EGFR, HER2, Mesothelin, Solid tumors

## Abstract

The chimeric antigen receptor T (CAR-T) cell therapy is a newly developed adoptive antitumor treatment. Theoretically, CAR-T cells can specifically localize and eliminate tumor cells by interacting with the tumor-associated antigens (TAAs) expressing on tumor cell surface. Current studies demonstrated that various TAAs could act as target antigens for CAR-T cells, for instance, the type III variant epidermal growth factor receptor (EGFRvIII) was considered as an ideal target for its aberrant expression on the cell surface of several tumor types. CAR-T cell therapy has achieved gratifying breakthrough in hematological malignancies and promising outcome in solid tumor as showed in various clinical trials. The third generation of CAR-T demonstrates increased antitumor cytotoxicity and persistence through modification of CAR structure. In this review, we summarized the preclinical and clinical progress of CAR-T cells targeting EGFR, human epidermal growth factor receptor 2 (HER2), and mesothelin (MSLN), as well as the challenges for CAR-T cell therapy.

## Background

**Fig. 1:**
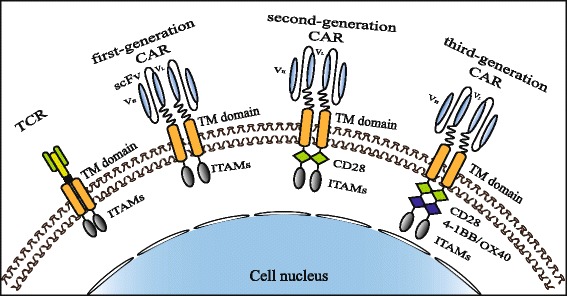
The structure of TCR and the three generations of CAR. T cell receptor (TCR) includes antigen-binding domain, transmembrane domain (TM domain), and immune receptor tyrosine-based activation motifs (ITAMs). The binding domain of CAR consists of a scFv, comprising the light (VL) and heavy (VH) variable fragments of a TAA-specific monoclonal antibody joined by a flexible linker. The intracellular parts are different between the three generations of CAR. The first-generation CAR only has the signal transduction domain of the CD3-zeta chain (CD3ζ) or Fc receptor γ (FcRγ) which mediated transient persistence, inefficient cytotoxicity, and low-level cytokine secretion. The second and third generation CAR add one or more co-stimulatory domains (CD28, 4-1BB, or OX40) to the first generation, which lead to the enhanced cytotoxicity and cytokine secretion along with prolonged T cell persistence

Over a century, immunology has been employed to treat malignant tumors, such as monoclonal antibody (mAb), bispecific antibody, tumor vaccine, immune checkpoint blockade, cytokine-induced killer (CIKs), tumor-infiltrating lymphocytes (TILs), and most recently chimeric antigen receptor T (CAR-T) [1]. Application of monoclonal antibodies (Herceptin, cetuximab) in malignant tumor patients showed a satisfying response rate. Immune checkpoint blockades are emerging immunotherapies against tumors. Pembrolizumab, nivolumab (anti-PD-1mAb), and ipilimumab (anti-CTLA-4mAb), which are representative immune checkpoint blocking agent, have been approved by the Food and Drug Administration (FDA) for melanoma patients, as either initial therapy or after relapse [2]. The CAR-T-based immunotherapy has achieved significant progress in malignant hematological diseases. CARs are synthetic receptors consisting of extracellular single-chain variable fragment (scFv), transmembrane domain, and intracellular part of immunoreceptor tyrosine-based activation motifs (ITAMs) and co-stimulatory signal (Fig. 1) [3]. The scFv is responsible for recognizing and binding to tumor-associated antigens (TAAs) expressed on the tumor cell surface. The endodomain plays a pivotal role in T cell activation, proliferation, persistence, and cytotoxicity. The structure of CAR is similar to T cell receptor (TCR), but the scFv of CAR recognizes TAAs independent of major histocompatibility complex (MHC) and targets a variety of antigens expressed on the surface of the tumor cell, including proteins, carbohydrates, and gangliosides (Fig. 1) [4, 5]. The first generation of CARs merely includes activation signal CD3 zeta chain (CD3ζ) or Fc receptor γ (FcRγ) in intracellular motif, thus inducing transient T cell activation [6]. The second and third generation of CARs including one activation domain and one or more costimulatory domains (CD28, 4-1BB, or OX40) were developed and contributed to the expansion, prolonged antitumor activity, and cytokine secretion (such as IL-2, TNFα, and IFN-γ) of T cell (Fig. 1) [7, 8]. Currently, anti-CD19 CAR-T cells were demonstrated to be effective in the treatment of B cell non-Hodgkin lymphoma (NHL), acute lymphoblastic leukemia (ALL), and chronic lymphocytic leukemia (CLL) [9–13]. Anti-CD116 has been developed for treating myelomonocytic leukemia [14].

Adoptive cellular therapy (ACT) using CAR-T cells is also a novel way for the treatment of other malignant tumors [15]. In solid tumors, epidermal growth factor receptor (EGFR), human epidermal growth factor receptor2 (HER2), carcinoembryonic antigen (CEA), disialoganglioside 2 (GD2), mesothelin, prostate-specific membrane antigen (PSMA), and interleukin-13Ra2 (IL13Ra2) are known as the targets of CAR-T cells. We summarized the current CAR-T cell-targeted antigens in Table 1. In this review, we mainly introduced the correlated studies of EGFR, HER2, and mesothelin-specific CAR-T cells. Those TAAs are commonly expressed on solid tumors and have been developed by multi-research institutes. More importantly, some studies have achieved promising outcome.

**Table 1:** Tumor-associated antigens of CAR-T cell target

Antigen	Full name	Disease
EGFR	Epidermal growth factor receptor	NSCLC, epithelial carcinoma, glioma
EGFRvIII	Variant III of the epidermal growth factor receptor	Glioblastoma
HER2	Human epidermal growth factor receptor 2	Ovarian cancer, breast cancer, glioblastoma, colon cancer, osteosarcoma, medulloblastoma
MSLN	Mesothelin	Mesothelioma, ovarian cancer, pancreatic adenocarcinoma
PSMA	Prostate-specific membrane antigen	Prostate cancer
CEA	Carcinoembryonic antigen	Pancreatic adenocarcinoma, breast cancer, colorectal carcinoma
GD2	Disialoganglioside 2	Neuroblastoma, melanoma
IL13Rα2	Interleukin-13Ra2	Glioma
GPC3	Glypican-3	Hepatocellular carcinoma
CAIX	Carbonic anhydrase IX	Renal cell carcinoma (RCC)
L1-CAM	L1 cell adhesion molecule	Neuroblastoma, melanoma, ovarian adenocarcinoma
CA125	Cancer antigen 125 (also known as MUC16)	Epithelial ovarian cancers
CD133	Cluster of differentiation 133 (also known as prominin-1)	Glioblastoma, cholangiocarcinoma (CCA)
FAP	Fibroblast activation protein	Malignant pleural mesothelioma (MPM)
CTAG1B	Cancer/testis antigen 1B (also known as NY-ESO-1)	Melanoma and ovarian cancer
MUC1	Mucin 1	Seminal vesicle cancer
FR-α	Folate receptor-α	Ovarian cancer

## Antitumor mechanism of CAR-T cells

**Fig. 2:**
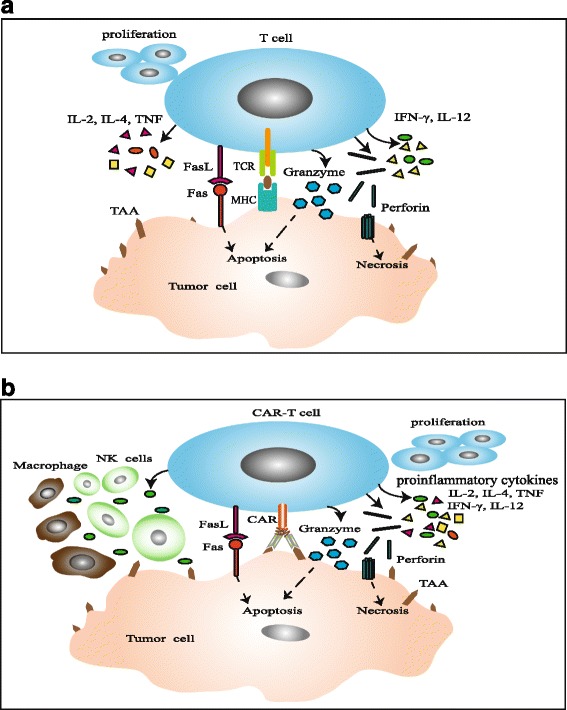
Antitumor mechanism of CAR-T. **a** TCR recognizes TAAs depending on the MHC presentation. The advantage is that TCR could recognize intracellular and extracellular antigens. While tumor cells often downregulate MHC expression to escape the killer T cells, **b** CAR-T cells can specifically recognize the tumor antigens in a MHC-independent manner. And then, the T cells were activated through the phosphorylation of ITAMs followed by enhanced cytokine (include IL-2, IL-4, IFN-γ, IL-12, and TNF) secretion, T cell proliferation, and cytotoxicity. IL-12 could recruit and reinforce the functions of innate immune cells such as NK cell and macrophage. Activated T and CAR-T cells perform cytotoxicity mainly through secretion of perforin and granzyme granules, also through the death receptor pathway such as Fas/Fas-L. Due to added co-stimulatory signal to endodomain, antitumor activity mediated by CARs is stronger than TCRs

CAR-T cells recognize specific tumor antigens in a MHC-independent manner, which lead to the activation and execution of its antitumor function [16]. Once CAR specifically binds with TAAs, T cells are activated through the phosphorylation of immune receptor tyrosine-based activation motifs (ITAMs) and subsequently induce cytokine secretion, T cell proliferation, and cytotoxicity [17]. The original T cells, including CD8^+^ and CD4^+^ T cells, are isolated from peripheral blood or tumor tissues of patients. It is generally agreed that CD8^+^ T cells play a critical part in immune responses against tumors, and CD4^+^ T cells can help to enhance the efficiency of CD8^+^ T cell-mediated cytotoxicity [18]. Chimeric immunoreceptor-activated T lymphocytes perform cytotoxicity through two predominant pathways: (1) secretion of perforin and granzyme granules and (2) activation of death receptor signaling via Fas/Fas-ligand (Fas-L) or TNF/TNF-R. CD8^+^ T cells kill tumor cells through those two pathways. CD4^+^ T cells destroy target cells primarily via perforin/granzyme, while death receptor-mediated apoptosis is believed to function as a compensatory pathway [19, 20]. Many strategies have been employed to potentiate the functions of CAR-T cells. It has been demonstrated that CAR-T cells with multiple signaling receptors could improve amplification, cytokine production, and cytotoxicity of T cells, as well as reduce antigen-induced cell death (AICD) in vitro and in vivo [21]. CD40L-modified T cells enhanced the proliferation and secretion of proinflammatory Th1 cytokines, including IL-2, IFN-γ, IL-12, and TNF [22]. CD28 costimulation was critical for antigen-specific cytokine secretion and T cell proliferation without obvious effect on the receptor-mediated target cell lysis [23]. IL-12 enhanced the activation of cytotoxic T cell [24], recruited and reinforced the functions of innate immune cells such as NK cell and macrophage [25], enhanced the Th1-type helper T cell response, and exhibited antiangiogenic activities [26]. On this basis, T cells redirected for universal cytokine killing (TRUCK) was developed. TRUCK is a way to redirect CAR-T cells by producing and releasing a transgenic product, such as IL-12, to activate innate immune response against tumor cells which are invisible to CAR-T cells [4]. Besides targeting antigen-specific tumor cell, IFN-γ secreted by CAR-T cells contributed to the antigen-independent destruction of tumor cell through IFNγR expressed in tumor stroma [27]. Neeson et al. developed a novel transgenic mouse model CAR OT-I. CAR OT-I cells not only recognized target tumor cells and secreted cytotoxic granule proteins (perforin, granzyme B) but also induced serial killing which were observed in real time via time-lapse microscopy [28]. In addition, the outcome of clinical application of CAR T cells could be improved by strengthening the function of CAR-T cells through co-activation of macrophage and NK cell (Fig. 2).

## Target antigen expressing on solid tumor cell surface

In this part, we summarized the preclinical and clinical studies of CAR-T antigens in solid tumors, focusing on the common targets of EGFR, HER2, and mesothelin. Emphases were put on the scientific basic and progress in preclinical experiments of CAR-T cells.

### EGFR

EGFR is a 170-KDa transmembrane receptor tyrosine kinase belonging to the ErbB (also known as HER) oncogene family [29–31]. EGFR is expressed in the skin, gastrointestinal system, kidney, and other normal tissues at the physiological level; however, it is aberrantly activated in many epithelial tumors, such as lung cancer, pancreatic cancer, colorectal cancer, breast cancer, and head and neck squamous cell carcinoma (HNSCC) [32, 33]. EGFR plays central roles in regulation of cellular multiplication, differentiation, and metastasis, and the overexpression of EGFR is related to a more aggressive clinical progression and poor prognosis [34, 35]. In fact, EGFR has been a therapy target for many years. Currently, targeted-EGFR antitumor agent is mainly divided into two categories: anti-EGFR monoclonal antibodies (mAbs) and small-molecule tyrosine kinase inhibitors (TKIs) [36]. Anti-EGFR mAbs prevent the EGF binding and receptor activation by occupying the ligand-binding site of the EGFR. TKIs inhibit autophosphorylation and downstream intracellular signaling of EGFR [37]. Two mAbs (cetuximab and panitumumab) and two TKIs (gefitinib and erlotinib), as first-generation EGFR inhibitors, have been used for the treatment of NSCLC, pancreatic cancer, HNSCC, renal cancer, and colorectal cancer (CRC) [38]. Nevertheless, the therapeutic efficacy of the EGFR inhibitors was attenuated in some patients, resulting from EGFR mutations and the acquired drug resistance. Hence, to create novel therapeutic strategies to overcome the defects is imperative [39, 40]. Anti-EGFR CAR-T therapy is an alternative strategy for EGFR overexpression malignant cancers, although the application of CAR-T therapy toward solid tumors remains challenging [41]. The most common oncogenic EGFR mutant is the type III EGFR (EGFRvIII), which results in an in-frame deletion of exons 2 to 7 [42, 43]. EGFRvIII appears to meet most of the criteria of ideal antigen for CAR-T therapy, for it is the most commonly altered form of EGFR in cancers with no expression in normal tissues [44]. Expression of EGFRvIII promotes tumor cell growth, invasion, migration, and therapeutic resistance and is associated with poor long-term survival [45, 46].

#### Preclinical studies on EGFR-specific CAR-T cells

Glioblastoma (GBM) remains one of the deadliest primary brain tumors in adults, and standard treatments for GBM do not significantly increase the survival time. EGFRvIII is expressed in GBM cell surface; therefore, CAR-T cell targeting EGFRvIII is a novel strategy worth studying [47]. Morgan et al. conducted a series of experiments to construct competent CARs and evaluated the ability of CAR-engineered T cells recognizing EGFRvIII. Considering that the established cell lines may not keep the molecular characteristics of primary human cancers, Morgan group selected the glioblastoma stem cells (GSCs) expressing EGFRvIII as target cell lines. CAR scFv derived from human mAb 139 recognized GSCs expressing mutant EGFRvIII, but not human normal tissues. T cell signaling transduction domain CD28-41BB-CD3ζ (named 139-28BBZ) made CAR-T keep better survival comparing with the original CAR vector that used CD28-CD3ζ (named 139-28Z) [48–50], but the biological activity and cytotoxicity were at the equal level. The engineered T cells expressing CAR can specifically recognize EGFRvIII+ cell lines, while no reactivity to co-cultured normal tissue cells. At present, a phase I clinical trial (NCT01454596) using anti-EGFRvIII CAR-T cells is recruiting patients with recurrent glioblastoma [51]. Study by Marcela et al. also evaluated the characteristics of anti-EGFRvIII CAR-T cells and verified its antitumor activity against glioblastoma cells in vitro and vivo [52]. The humanized anti-EGFRvIII CAR-T cells produced IFN-γ, IL-2, TNF-α, and only lysed EGFRvIII-expressing target cells. In order to confirm the antitumor activity in vivo, U87-EGFRvIII tumors were implanted subcutaneously and intracranially into NSG mice, respectively. The results indicated that CAR-T-EGFRvIII cells controlled tumor growth and increased median survival time. This group also used mice grafted with normal human skin to test the potential toxicities of anti-EGFRvIII CAR-T cells, and the results of skin graft assay demonstrated that no significant lymphocytic infiltrate by immunohistochemistry. On this basis, Marcela group started a phase 1 clinical trial (NCT02209376) of EGFRvIII-specific CAR-T cells in patients with either residual or recurrent glioblastoma [53]. D-270MG is a tumor cell line that naturally expresses EGFRvIII [54]. Sampson et al. established the D-270MG^FLuc/GFP^ subline that co-expressed firefly luciferase (FLuc) and GFP as the target of EGFR-specific CAR-T cells. The study results demonstrated that anti-EGFRvIII CAR-T cells effectively transpassed the blood-brain barrier (BBB) to arrive at invasive GBM tumors and mediated tumor regression and prolonged survival in NSG mice [55]. Zuo et al. used the EGFR-positive (EGFR+) cells including A549, NCI-H1299, NCI-H460, SGC7901, HT29, and EGFR-knockdown (EGFR−) cells including A549-EGFR−, SGC7901-EGFR−, and HT-29-EGFR− to investigate the antitumor activity of EGFR-specific CAR-CIK cells. The study reported that EGFR-specific CAR observably potentiated cytotoxicity and induced secretion of IFN-γ and IL-2 in EGFR-positive cell lines and xenograft tumor models, but not in EGFR negative ones [56]. In summary, the preclinical studies of EGFR-specific CAR-T cells exhibited potent antitumor effect in vitro and in vivo.

#### Clinical trials on EGFR-specific CAR-T cells

Multicenter clinical trials using CAR-T cells targeting EGFR or EGFRvIII are underway. We summarized these clinical trials in Table 2. A phase I trial by Han et al. studied the EGFR-targeted CAR-T cells in 11 patients with EGFR-expressing advanced relapsed/refractory NSCLC (NCT01869166). In this study, the six female and five male patients were divided into three cohorts: in cohort 1, EGFR-CAR T cells infused into four patients directly without any conditioning regimens; in cohort 2, two patients were conditioned with cyclophosphamide, followed by CAR-T EGFR therapy; and in cohort 3, two patients were conditioned by cyclophosphamide, pemetrexed, and cisplatin and three were conditioned by cyclophosphamide, docetaxel, and cisplatin, respectively. All patients received EGFR-targeted CAR-T cell infusions at dose ranged from 0.45 to 1.09 × 10^7^ cells/kg. Of 11 patients, there were two persons acquired PR and five kept stable disease (SD). The anti-EGFR CAR-T cells secreted cytokines including IL-2, IL-4, IL-6, IFN-γ, TNF-α, GM-CSF, and granzyme B in co-culture with EGFR-positive tumor cells. However, after the infusion of EGFR-specific CAR-T cells, the serum levels of cytokines observed at different time point were less obvious compared with the experiment in vitro. Investigators monitored the copy numbers of CAR-EGFR transgene in peripheral blood (seven patients) and tumor tissues (four patients) by quantitative real-time PCR. In peripheral blood, the copy numbers of CAR-EGFR transgene hold high level for more than 4 weeks. CAR-EGFR transgene specifically accumulated in tumor tissues. The only tolerable and controllable toxicities reported in the study were skin toxicity, nausea, vomiting, dyspnea, and hypotension, and there was no cytokine storm observed. Therefore, the CAR-T-EGFR cells were found to be feasible and safe in patients of relapsed/refractory NSCLC [57].

**Table 2:** Clinical trials of CAR-T cells

Target	Identifier	Institution	Phase	Status	Disease	Comments
EGFR	NCT02331693	Shanghai Cancer Institute	I	Recruiting	Glioma	Autologous T cells transduced with a lentiviral vector
EGFRvIII	NCT02844062	Beijing Sanbo Brain Hospital	I	Recruiting	Glioma	Lymphodepletion chemotherapy, followed by CAR-T
EGFRvIII	NCT01454596	National Cancer Institute (NCI)	I/II	Recruiting	Glioma	Autologous T cells, a retroviral vector
EGFRvIII	NCT02209376	University of Pennsylvania/University of California	I	Recruiting	Glioma	Autologous T cells, a lentiviral vector
EGFRvIII	NCT02664363	Duke University Medical Center	I	Not yet recruiting	Glioma	Dose escalation cohorts for 4 dose levels
EGFR	NCT01869166	Chinese PLA General Hospital	I/II	Completed	NSCLC	Safe and feasible
HER2	NCT02713984	Southwest Hospital, China	I/II	Recruiting	HER2-positive cancer	
HER2	NCT01935843	Chinese PLA General Hospital	I/II	Recruiting	HER2-positive cancer	
HER2	NCT02547961	Fuda Cancer Hospital Guangzhou	I/II	Recruiting	Breast cancer	A retrovirus vector, preconditioning treatment
HER2	NCT02442297	Baylor College of Medicine	I	Recruiting	Glioma	Intracranial injection
HER2	NCT01109095	Baylor College of Medicine	I	Active, not recruiting	Glioma	CMV-specific cytotoxic T cells (CMV-T cells)
HER2	NCT00889954	Baylor College of Medicine	I	Active, not recruiting	HER2-positive cancer	TGFBeta resistant HER2/EBV-CTLs
HER2	NCT00924287	National Cancer Institute (NCI)	I/II	Completed	HER2-positive sarcoma	Results is not encouraging
HER2	NCT00902044	Baylor College of Medicine	I/II	Completed	HER2-positive sarcoma	Safe and feasible
MSLN	NCT02930993	China Meitan General Hospital	I	Recruiting	Mesothelin-positive tumors	Followed lymphodepletion
MSLN	NCT02706782	Shanghai Renji Hospital	I	Recruiting	Pancreatic cancer	Transcatheter arterial infusion
MSLN	NCT02159716	University of Pennsylvania	I	Active not recruiting	Mesothelin-positive tumors	Lentiviral transduced
MSLN	NCT02792114	Memorial Sloan Kettering Cancer Center	I	Recruiting	Mesothelin-expressing breast cancer	Premedicated with acetaminophen and diphenhydramine, and administered cyclophosphamide
MSLN	NCT02465983	University of Pennsylvania	I	Active not recruiting	Pancreatic cancer	Combination therapy with CART-meso cells and CART19 cells
MSLN	NCT02959151	Shanghai Tumor Hospital	I/II	Recruiting	Pancreatic cancer	Combined with interventional therapy
MSLN	NCT02590747	Chinese PLA General Hospital	I	Recruiting	Mesothelin-positive tumors	Retroviral vector-transduced
MSLN	NCT01355965	University of Pennsylvania	I	Completed	Pleural mesothelioma	Safe and feasible
MSLN	NCT02414269	Memorial Sloan Kettering Cancer Center	I	Recruiting	Malignant pleural disease	With/without chemotherapy
MSLN	NCT01583686	National Cancer Institute (NCI)	I/II	Recruiting	Mesothelin-positive tumors	Followed lymphodepletion
MSLN	NCT01897415	University of Pennsylvania	I	Active not recruiting	Pancreatic ductal adenocarcinoma (PDA)	Transfected with chimeric anti-mesothelin immunoreceptor SS1
IL13Rα2	NCT00730613	City of Hope Medical Center	I	Completed	Glioblastoma	Safe and feasible
IL13Rα2	NCT02208362	City of Hope Medical Center	I	Completed	Glioblastoma	Safe and feasible
CEA	NCT01373047	Roger Williams Medical Center	I	Completed	Liver metastases	Safe and feasible
FAP	NCT01722149	University of Zurich	I	Recruiting	Malignant pleural mesothelioma	Followed lymphodepletion
GD2	NCT00085930	Baylor College of Medicine	I	Completed	Neuroblastoma	Safe and feasible
GD2	NCT02107963	National Cancer Institute (NCI)	I	Completed	Sarcoma Osteosarcoma Neuroblastoma Melanoma	Safe and feasible
CD133	NCT02541370	Chinese PLA General Hospital	I	Recruiting	CD133-positive malignancies	Relapsed and/or chemotherapy refractory advanced malignancies

The details of Table 2 derived from http://clinicaltrials.gov/

### HER2

HER2 is a 185-KDa transmembrane glycoprotein which also belongs to the family of the EGFR [58, 59]. *HER2* gene amplification or HER2 overexpression plays a crucial role in the biologic behavior and pathogenesis of some type of human cancers [60]. HER2 is overexpressed in 25–30% of breast and ovarian cancers [61], up to 60% of human osteosarcomas (OS) [62], approximately 80% of GBM [63], and 40% of medulloblastomas but is not detected in normal cerebellum and other brain tissues [64]. Overexpression of HER2 is associated with cellular transformation and carcinogenesis and also correlated with poor clinical outcome [65, 66]. On this basis, HER2 monoclonal antibody trastuzumab (Herceptin) was first approved for use in patients with HER2-overpressed breast cancer. Trastuzumab alone or in combination with chemotherapy prolongs survival in both primary and metastatic breast cancer [67]. At present, the clinical trials about HER2 tyrosine kinase inhibitors such as lapatinib and neratinib are still ongoing [68]. However, many tumors such as osteosarcoma, glioblastoma, and medulloblastoma expressing HER2 at low levels are ineffectively recognized by trastuzumab [66]. In addition, approximately half of those patients either do not respond to these therapies or develop secondary resistance which results to treatment failure [69, 70]. Therefore, it is necessary to create novel therapeutic approach to treat these patients.

#### Preclinical studies on HER2-specific CAR-T cells

In GBMs, CD133-positive stem cells keep higher expression of HER2 than CD133-negative counterparts. A study result indicated that HER2-specific CAR-T cells targeted and killed autologous HER2-positive GBMs in vitro and facilitated regression of GBMs in an orthotopic xenograft model [71]. Sun et al. constructed a humanized HER2 CAR-T cell containing chA21scFv and examined its antitumor activity. The results indicated that chA21-28z HER2-specific CAR-T cells recognized and killed HER2+ breast and ovarian cancer cells in vitro. Simultaneously, abundant IFN-γ and IL-2 secretion were also detected. In xenograft model, the HER2-specific CAR-T cells also significantly restricted tumor growth [72]. Another study demonstrated that oligoclonal camelid single-domain antibodies (VHHs) could target a range of different epitopes on HER2 antigen. Based on the potent targeting ability of oligoclonal VHHs, the oligoclonal VHH_HER2_-CAR-engineered Jurkat T cells exhibited higher expansion, cytokine secretion, and cytotoxicity when exposed to HER2-expressing cells [73]. To reduce antigen escape, Hegdeet et al. created a bispecific CAR molecule co-targeting the two glioma-associated antigens, HER2 and IL-13Rα2, and expanded the CAR-T cells expressing tandem CARs (TanCAR). Encouragingly, the TanCAR effectively redirected T cells to the two antigens and enhanced the function of CAR-T cells and the secretion of cytokines in vitro and in vivo. Therefore, the TanCAR-T cell agents were considered as a potential therapeutic method to control tumor growth as this study reported [74, 75]. Recently, a group combined bispecific antibody αHER2/CD3 and CAR-T therapy. Their data indicated that αHER2/CD3 RNA-engineered T cells exhibited antitumor activity in HER2^+^ N87 tumor cells and in N87 tumor-bearing mice. Moreover, bystander T cells also showed the similar effects. This new strategy may be a potential therapeutic approach for HER2^+^ malignancies [76]. To promote the transduction efficiency, EBV-CTLs were modified to express HER2-CAR via the nonviral piggyBac (PB) transposon which had high gene-transfer efficiency and large coding capacity. PB-modified HER2-CTLs could specifically target and kill HER2-positive tumor cells in vivo and suppress tumor growth in xenogeneic murine models [77]. Although 60% human osteosarcoma expressed HER2 [62, 78], a low level of HER2 renders monoclonal antibodies to HER2 ineffective. Hence, a group used genetic-modified T cell targeting HER2 to determine the antitumor activity in osteosarcoma. The HER2-specific CAR-T cells proliferated, produced cytokines, and killed tumor cells after exposure to HER2-positive osteosarcoma cell lines in vitro. Moreover, they created two mouse models: one is locoregional disease in a severe combined immune deficiency (SCID) mouse model and the other is lung metastases model. Adoptive transfer of HER2-specific CAR-T cells caused osteosarcoma regression at the different sites [79]. Similarly, HER2-specific CAR-T cells had the capacity of recognizing and killing HER2-positive medulloblastoma cells in vitro and induced regression of tumors in an orthotopic xenogeneic SCID model [64]. These preclinical studies have achieved encouraging results, promoting HER2-specific CAR-T clinical trials to test the feasibility and safety.

#### Clinical trials on HER2-specific CAR-T cells

At present, Southwest Hospital in China, Chinese PLA General Hospital, Fuda Cancer Hospital Guangzhou, and Baylor College of Medicine are carrying out clinical trials of HER2-specific CAR-T cells. We summarized these clinical trials in Table 2. Phase I/II clinical study (NCT00924287) sponsored by National Cancer Institute (NCI) has completed. This trial was designed to evaluate the safety and efficacy of HER2-specific CAR-T cells in patients with relapsed/refractory HER2-positive sarcoma. Nineteen patients received escalating doses (range 1 × 10^4^/m^2^ to 1 × 10^8^/m^2^) of HER2-specific CAR-T cells including eight dose levels. The study reported that among the detected serum cytokines, only the concentration of IL-8 had significantly increased within 1 week after infusion and persisted for up to 4 weeks. Although HER2-specific CAR-T cells had no expansion after infusion in the peripheral blood, these cells could traffic to tumor sites and maintain at low levels for more than 6 weeks. T cell persistence and copy number were correlated with the infused T cell dose. The clinical benefit of HER2-specific CAR-T cell was not encouraging, only four of nineteen patients acquired stable disease (SD). In the process of HER2-specific CAR-T cell infusion, dose-limiting toxicity was not observed apart from a patient with the highest dose levels within 12 h post-infusion [80].

### Mesothelin

Mesothelin (MSLN) is a 40-KDa cell surface tumor differentiation antigen, which derived from the 69-KDa precursor protein encoded by *Mesothelin* gene [81, 82]. The normal biological function of mesothelin almost remains unknown. Some studies suggest that mesothelin is the receptor of CA125/MUC16, and the interaction between mesothelin-CA125 mediates cell adhesion and may be a critical point in the metastatic of ovarian cancer [83, 84]. Mesothelin overexpression promotes tumor cell proliferation and regional invasion and is associated with poor prognosis, such as worse recurrence-free survival (RFS) and overall survival (OS) [85–87]. As a tumor marker, soluble mesothelin in serum plays an important role in diagnosing and monitoring therapeutic effect for patients with malignant pleural mesothelioma (MPM) and ovarian cancer [88–91]. Mesothelin is expressed at low levels in normal tissues, including pleura, pericardium, peritoneum, tunica vaginalis [92–94], but it is overexpressed in various malignancies including MPM, ovarian cancers, pancreatic cancers, and non-small cell lung cancers [95–98]. Due to the weak expression in normal tissues and strong expression in several cancers, mesothelin is considered as an attractive target for immune-based therapies [81]. With respect to mesothelin-targeted therapies such as anti-mesothelin recombinant immunotoxin SS1P, chimeric anti-mesothelin monoclonal antibody MORAb-009, and mesothelin cancer vaccines CRS-207, investigators performed a lot of preclinical researches and opened a series of clinical trials [99–102]. Simultaneously, a number of studies about CAR-T cells targeting mesothelin are in progress.

#### Preclinical studies on MSLN-specific CAR-T cells

June et al. demonstrated that the mesothelin-specific T cells exhibit antitumor effects on large pre-established mesothelioma xenografts in NOD/scid/IL2rγ^−/−^ mice. Their data suggested that the combination of CD137 and CD28 improved multifunctional cytokine secretion and enhanced the function of CAR T cells in tumor-bearing mice [103]. In the tumor microenvironment, some inhibitors hampered the function of CAR T cells. For example, diacylglycerol kinase (dgk), as a negative regulator of TCR signaling, is expressed in T cells. Its isoform includes dgkα and dgkζ. Previous studies found that deletion of either dgk isoform induced the activation of DAG-mediated Ras/ERK pathway and proliferation of T cells [104–106]. Based on this, Koretzky et al. demonstrated that deletion of dgks greatly enhanced activity against tumor and improved persistence of CAR-engineered T cells targeting mesothelin in vitro and in implanted tumors. Beyond that, pharmacologic inhibition of dgks also facilitated function of mesothelin-specific CAR-T cells. Moreover, dgk-deficient T cells showed decreased sensitivity to TGFβ and increased FasL and TRAIL expression. Such a combined therapeutic approach might be translated clinically as the study reported [107]. Moon et al. found that a single intravenous injection of human mesoCAR-T cells into immunodeficient mice significantly restrained the tumor growth but did not cure tumor. They considered that upregulation of inhibitory receptors was the main cause of mesoCAR-T cell hypofunction [108]. As an inhibitor within the tumor microenvironment, upregulation of PD-1 limited T cell function [109]. Cherkassky et al. found that PD-1 antibody could reverse PD-1-mediated CAR-T cell exhaustion and mesoCAR-T cells also showed delayed exhaustion upon repeated antigen stimulation. Hence, combination of costimulation and cell-intrinsic PD-1 checkpoint blockade could overcome inhibitory effect on CAR-T cells in MSLN-expressing tumor microenvironment [110]. CAR-T therapy achieved good results in preclinical studies. But the effect was not satisfied in the clinical trials mainly due to its adverse effects. For example, scFv was generally derived from murine monoclonal antibodies; the induction of human anti-mouse antibody (HAMA) might shorten T cell survival time [111]. A study demonstrated that fully human mesothelin-specific CAR-T cells showed potent cytolytic activity toward mesothelin-positive tumor cells and controlled large, well-established ovarian cancer growth in a xenogeneic mouse model. Besides, mesothelin-specific CAR-T cells induced bystander killing of mesothelin-negative tumor cells [112]. On-target/off-tumor toxicity could cause life-threatening adverse effects in the application of CAR-T cells, because the target antigen also expressed on normal cell surface at low levels. Both a-folate receptor (FRa) (90%) and mesothelin (70%) were overexpressed in ovarian cancers [113, 114], and their expression pattern on normal tissues is mainly non-overlapping. Based on the foundation of above studies, Daniel et al. generated trans-signaling CAR T cells engineered to co-express anti-mesoscFv-CD3 and anti- FRascFv-CD28CARs, aiming to diminish the potential toxicity of CAR-T cells to normal tissue cells expressing low levels of TAAs. The result indicated that trans-signaling CAR-T cells exhibited higher antitumor potential in vitro and in vivo. Moreover, trans-signaling CAR-T cells were resistant to antigen-induced cell death (AICD) [115]. The successes achieved by CAR-T cells in hematological malignancies were unable to be accomplished in solid tumor, partly owing to the low efficacy of CAR-T cells homing to tumor sites. Stimulating more chemokine receptors expressed on CAR-T cells or direct regional injection may be valid. Chemokine CCL2 is highly expressed by MPM tumors, but the expression level of CCL2 receptor CCR2 on resting and activated T cells is low. Therefore, Moon et al. transduced the chemokine receptor CCR2b into mesoCAR-T cells to potentiate trafficking of CAR-T cells into tumors. Their study demonstrated that the functional CCR2b in the mesoCAR-T cells significantly increased the number of intratumoral T cells and improved antitumor efficacy in vitro and in vivo [116]. Adusumilli et al. found that compared with intravenous injection, intrapleural administration of anti-mesothelin CAR-T cells exhibited greater antitumor potency and strongly promoted the expansion, differentiation, and persistence of T cells [117].

#### Clinical trials on MSLN-specific CAR-T cells

Many clinical trials about mesothelin-specific CAR-T cells are ongoing. We summarized these clinical trials in Table 2. Marcela et al. started a clinical study in four patients infused with autologous T cells transducted with mRNA to express CAR derived from a murine antibody to human mesothelin. These results demonstrated that when patients received intermittent infusion of meso-RNA CAR-T cells, the serum IgE levels detected via ELISA assay were elevated which caused anaphylaxis. Therefore, they suggested that a single infusion of stably transducted, long-lived CAR-T cells or constructing CAR based on the humanized antibodies may be safer and more effect [52]. The phase I clinical trial (NCT01355965) conducted by Beatty et al. was designed to improve the feasibility and safety of mRNA-transduced CAR-T cells targeting mesothelin (mesoCAR-T cells) in patients with advanced MPM. They presented two case reports indicating that mRNA CAR-T cells showed potent antitumor activity without evident on-target/off-tumor toxicity against normal tissues, infiltrated solid tumor tissues, and induced humoral epitope spreading after infusion [118].

### Other target antigens

In addition, there are lots of tumor-associated antigens studied by investigators in solid tumors. CA125 also called MUC16 is a well-known ovarian tumor antigen routinely used for monitoring disease. To enhance the antitumor efficacy, Brentjens et al. developed T cells co-expressing MUC16 CAR and IL-12, and the results were as expected both in vitro and in vivo [119]. Based on the rationale, they opened a phase I clinical trial in patients with recurrent ovarian cancer [120]. Carbonic anhydrase IX (CAIX) is an attractive target antigen because it is overexpressed in renal cell carcinoma (RCC) but is not found on normal kidney tissue. The CAIX-specific CAR-T cells inhibited tumor growth in xenograft model [121]. Several malignant tumors including pancreatic adenocarcinoma, breast cancer, and colorectal carcinoma overexpressed carcinoembryonic antigen (CEA). Guest et al. generated CAR-T cells for the phase I/II clinical trial of CEA-specific CAR-T therapy in 14 patients with advanced CEA+ malignancy [122]. At present, clinical trials of anti-CEA CAR-T cells in Advanced Liver Malignancy (NCT02959151) and CAR-T Cells Targeting CEA Positive Cancer (NCT02349724) are ongoing. Neuroblastoma is a high-risk extracranial malignant tumor of childhood. Disialoganglioside (GD2) is overexpressed in almost all neuroblastoma. Therefore, GD2 is an ideal candidate of CAR-T cells. The preclinical and clinical studies of GD2-specific CAR-T cells have achieved some progress [123, 124]. Moreover, the clinical trial of GD2-specific CAR-T therapy in 19 patients with advanced neuroblastoma has completed by Louis et al. It was showed that eight achieved remission and 11 with active disease [125]. A study reported that the GD2-specific CAR-T cells showed anti-melanoma activity in vitro and in vivo [126]. Similar to GD2, L1 cell adhesion molecule (L1-CAM) is also overexpressed in neuroblastoma. In addition, ovarian adenocarcinoma, medulloblastoma, and melanoma all highly expressed L1-CAM [127]. Investigators tested the antitumor efficacy and safety in preclinical and clinical studies [128–130]. Glypican 3 (GPC3) is highly expressed in hepatocellular carcinoma (HCC) and hepatoblastoma. Study results demonstrated that all GPC3-CAR-T cells showed potent cytotoxicity to GPC3-positive cells [131]. Aiming at GPC3 and asialoglycoprotein receptor1 (ASGR1) another TAA in HCC, a group developed the dual-targeted CAR-T cells. They found that dual-targeted CAR-T cells caused higher proliferation, antitumor activity, and cytokine secretion than signal-targeted CAR-T cells in vitro [132]. Prostate-specific membrane antigen (PSMA) was expressed in prostate cancer cells. PSMA-targeted CAR-T cells exhibited superior antitumor efficacy in vitro. In established models, PSMA-targeted CAR-T cells also effectively eliminated prostate cancer [133–135]. CD133, as a specific molecular biomarker for CSCs, is an attractive therapeutic target for CAR-T therapy [136, 137]. CD133-specific CAR-T cells in a patient with advanced cholangiocarcinoma have shown antitumor activity [138]. At present, a phase I clinical trial of anti-CD133 CAR-T cells in patients with relapsed and/or chemotherapy refractory advanced malignancies is ongoing (NCT02541370). In addition to above antigens, fibroblast activation protein (FAP) [139, 140], NY-ESO-1 [141], MUC1 [142], foliate receptor [143, 144], and IL13Rα2 [145, 146] are also potential target antigens for immunotherapy.

## Conclusions

In this review, we summarized the current preclinical and clinical studies on CAR-T therapy against solid tumors, especially targeting EGFR, HER2, and MSLN. The ideal target for CAR-T cells would be the tumor-specific antigens which are homogenously expressed on the surface of malignant cell and play a critical role in tumorigenesis. Although the curative effect in CAR-T treatments of hematological malignancies are reported, the results of pilot clinical trials on solid cancers are below expectation. Several obstacles have remained to be overcome for a successful application of CAR-T cells in solid tumor, including the lack of ideal TAAs, inefficient trafficking of CAR-T cells to tumor sites, hostile solid tumor microenvironment, and the risk of developing on-target/off-tumor toxicities [15, 17]. To solve the problems, investigators have developed some strategies to potentiate the trafficking of CAR-T cells [116], reduce the inhabitation effect of tumor microenvironment [110], decrease the adverse effects, and so on [115]. In general, the preclinical studies of CAR-T cells in vitro and in vivo showed potent antitumor efficacy; with further exploration to improve the feasibility, safety, and efficiency of CAR-T cells, CAR-T therapy will take the central stage in the treatment of solid tumors.

## Abbreviations

ACT: Adoptive cellular therapy
AICD: Antigen-induced cell death
ALL: Acute lymphoblastic leukemia
ASGR1: Asialoglycoprotein receptor 1
CAIX: Carbonic anhydrase IX
CARs: Chimeric antigen receptors
CD3ζ: CD3 zeta chain
CEA: Carcinoembryonic antigen
CIKs: Cytokine-induced killer
CLL: Chronic lymphocytic leukemia
CRC: Colorectal cancer
dgk: diacylglycerol kinase
EGFR: Epidermal growth factor receptor
EGFRvIII: Type III variant epidermal growth factor receptor
Fas-L: Fas-ligand
FcRγ: Fc receptor γ
FDA: Food and Drug Administration
GBM: Glioblastoma
GD2: Disialoganglioside
HAMA: Human anti-mouse antibody
HCC: Hepatocellular carcinoma
HER2: Human epidermal growth factor receptor2
HNSCC: Head and neck squamous cell carcinoma
IL13Ra2: Interleukin-13Ra2
ITAMs: Immunoreceptor tyrosine-based activation motifs
mAb: Monoclonal antibody
MHC: Major histocompatibility complex
MPM: Malignant pleural mesothelioma
MSLN: Mesothelin
NCI: National Cancer Institute
NHL: Non-Hodgkin lymphoma
NK: Natural killer
OS: Osteosarcomas
PB: PiggyBac
PSMA: Prostate-specific membrane antigen
RCC: Renal cell carcinoma
RFS: Recurrence-free survival
scFv: Single-chain variable fragment
SCID: Severe combined immune deficiency
SD: Stable disease
TAAs: Tumor-associated antigens
TanCAR: Tandem CARs
TCR: T cell receptor
TILs: Tumor-infiltrating lymphocytes
TKIs: Tyrosine kinase inhibitors
TRUCK: T cells redirected for universal cytokine killing
